# Supplementary material for: Distinct Gene Expression Profiles of Matched Primary and Metastatic Triple-Negative Breast Cancers
Source: Cancers (Basel). 2022 May 16;14(10):2447. doi: 10.3390/cancers14102447 (PMC9139196; doi:10.3390/cancers14102447)
Supplement: Supplementary file 1 [file cancers-14-02447-s001.zip › cancers-1720695-supplementary.pdf]

Supplementary Materials

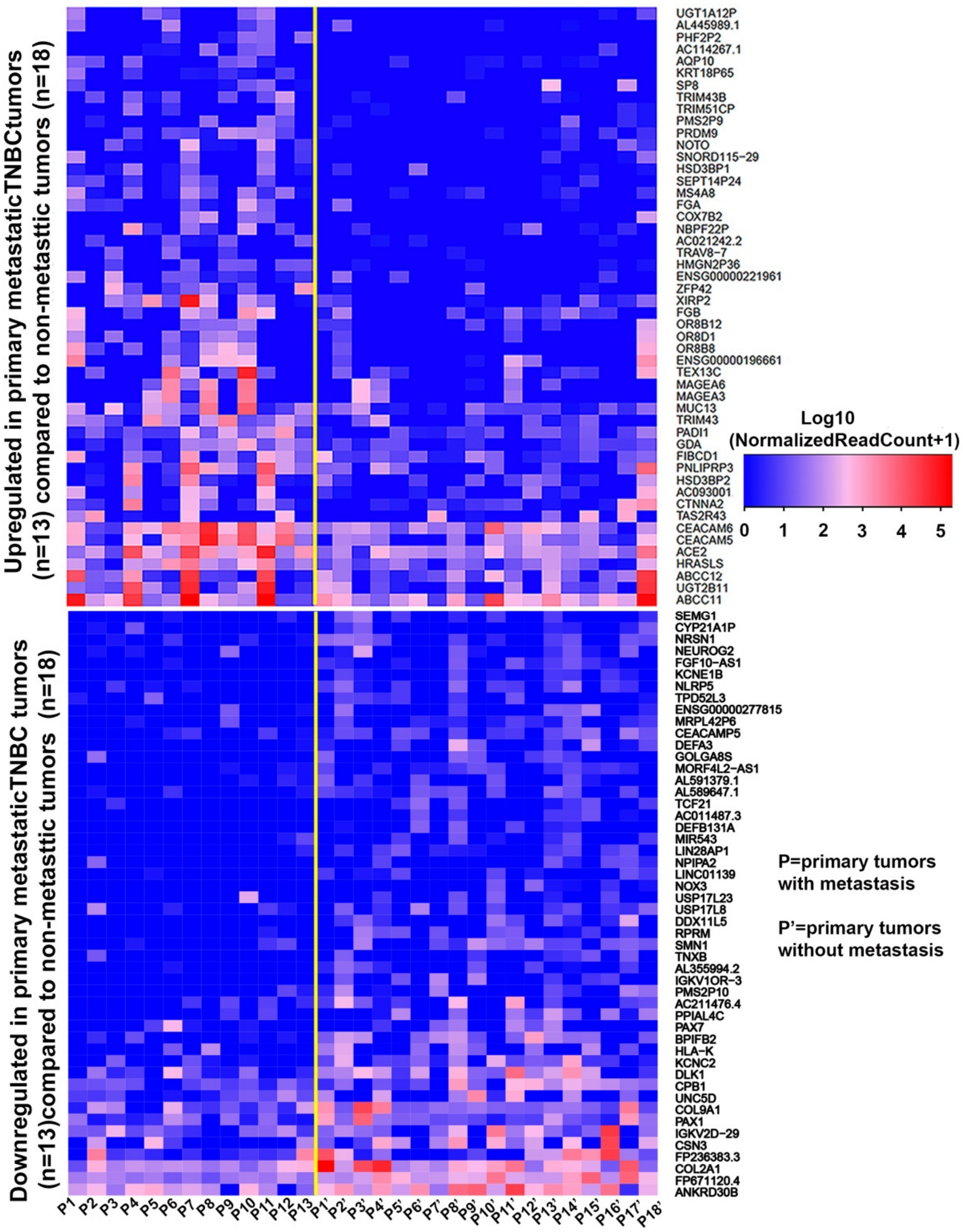

Supplementary Figure S1. DEGs between primary metastatic and non-metastatic TNBC tumors. Heatmap showing the top 100 upregulated and downregulated genes in primary TNBC tumors with metastasis (P; n=13) compared to primary TNBC tumors without metastasis (P'; n=18) in the Zurich cohort.

Supplementary Table S1. GO biological processes enriched among DEGs in the Zurich cohort.

GO ID	Biological Processes	*P*-Value	No. of Genes
GO:0002250	Adaptive immune response	1.00E-20	36
GO:0050776	Regulation of immune response	3.10E-16	34
GO:0050853	B cell receptor signaling pathway	4.22E-14	19
GO:0006955	Immune response	5.81E-13	47
GO:0031295	T cell costimulation	4.07E-10	18
GO:0042102	Positive regulation of T cell proliferation	5.61E-09	15
GO:0045087	Innate immune response	8.19E-09	40
GO:0006958	Complement activation, classical pathway	1.90E-08	18
GO:0007166	Cell surface receptor signaling pathway	9.36E-08	29
GO:0042110	T cell activation	2.38E-07	12
GO:0050852	T cell receptor signaling pathway	1.57E-06	19
GO:0042113	B cell activation	2.82E-06	9
GO:0034765	Regulation of ion transmembrane transport	3.10E-06	16
GO:0042100	B cell proliferation	6.35E-06	9
GO:0006911	Phagocytosis, engulfment	1.30E-05	9
GO:0007169	Transmembrane receptor protein tyrosine kinase signaling pathway	1.37E-05	14
GO:0045060	Negative thymic T cell selection	1.41E-05	6
GO:0050871	Positive regulation of B cell activation	1.44E-05	8
GO:0030168	Platelet activation	2.23E-05	15
GO:0006956	Complement activation	2.42E-05	13
GO:0006910	Phagocytosis, recognition	2.44E-05	8
GO:0030574	Collagen catabolic process	3.86E-05	11
GO:0006954	Inflammatory response	5.08E-05	29
GO:0042742	Defense response to bacterium	7.87E-05	16
GO:0007204	Positive regulation of cytosolic calcium ion concentration	0.000122	15
GO:0086010	Membrane depolarization during action potential	0.000238	7
GO:0038096	Fc-gamma receptor signaling pathway involved in phagocytosis	0.000258	14
GO:0030449	Regulation of complement activation	0.000354	7
GO:0007165	Signal transduction	0.000445	60
GO:0045086	Positive regulation of interleukin-2 biosynthetic process	0.000447	5
GO:0006959	Humoral immune response	0.000488	9
GO:0009967	Positive regulation of signal transduction	0.000776	9
GO:2000147	Positive regulation of cell motility	0.000859	5
GO:0032735	Positive regulation of interleukin-12 production	0.001114	6
GO:0030183	B cell differentiation	0.001314	9
GO:0030049	Muscle filament sliding	0.001321	7
GO:0050862	Positive regulation of T cell receptor signaling pathway	0.001703	4
GO:0038083	Peptidyl tyrosine autophosphorylation	0.001739	7

GO:0002407	Dendritic cell chemotaxis	0.00189	5
GO:0043066	Negative regulation of apoptotic process	0.002083	28
GO:0006936	Muscle contraction	0.002573	11
GO:0030217	T cell differentiation	0.002613	6
GO:0051712	Positive regulation of killing of cells of other organism	0.003109	3
GO:0035556	Intracellular signal transduction	0.003434	25
GO:0001771	Immunological synapse formation	0.003476	4
GO:0035115	Embryonic forelimb morphogenesis	0.003501	6
GO:0032753	Positive regulation of interleukin-4 production	0.003561	5
GO:0032729	Positive regulation of interferon-gamma production	0.0036	7
GO:0071222	Cellular response to lipopolysaccharide	0.003826	11
GO:0006968	Cellular defense response	0.00391	8
GO:0007155	Cell adhesion	0.004626	27
GO:0030198	Extracellular matrix organization	0.005026	15
GO:0007268	Chemical synaptic transmission	0.005512	17
GO:0030595	Leukocyte chemotaxis	0.006071	4
GO:0071353	Cellular response to interleukin 4	0.007047	5
GO:0006957	Complement activation, alternative pathway	0.007703	4
GO:0030890	Positive regulation of B cell proliferation	0.008309	6
GO:0072540	T-helper 17 cell lineage commitment	0.009921	3
GO:0033153	T cell receptor V(D)J recombination	0.009921	3
GO:0042391	Regulation of membrane potential	0.01101	8
GO:0019228	Neuronal action potential	0.012258	5
GO:0035116	Embryonic hindlimb morphogenesis	0.012258	5
GO:0038095	Fc-epsilon receptor signaling pathway	0.013848	13
GO:0001768	Establishment of T cell polarity	0.014563	3
GO:0046632	Alpha-beta T cell differentiation	0.014563	3
GO:2000107	Negative regulation of leukocyte apoptotic process	0.014563	3
GO:0070588	Calcium ion transmembrane transport	0.015801	10
GO:0006814	Sodium ion transport	0.016351	8
GO:0045860	Positive regulation of protein kinase activity	0.01792	6
GO:0006898	Receptor-mediated endocytosis	0.01895	13
GO:0045954	Positive regulation of natural killer cell-mediated cytotoxicity	0.019483	4
GO:0050869	Negative regulation of B cell activation	0.019952	3
GO:0002606	Positive regulation of dendritic cell antigen processing and presentation	0.019952	3
GO:0072678	T cell migration	0.019952	3
GO:0042832	Defense response to protozoan	0.022588	4
GO:0007157	Heterophilic cell-cell adhesion via plasma membrane cell adhesion molecules	0.022876	6
GO:0050850	Positive regulation of calcium-mediated signaling	0.025945	4
GO:0048304	Positive regulation of isotype switching to IgG isotypes	0.026034	3
GO:0034695	Response to prostaglandin E	0.026034	3
GO:0007160	Cell-matrix adhesion	0.027456	8

GO:0048469	Cell maturation	0.028785	5
GO:0019835	Cytolysis	0.029555	4
GO:0050764	Regulation of phagocytosis	0.03276	3
GO:0046641	Positive regulation of alpha-beta T cell proliferation	0.03276	3
GO:0090023	Positive regulation of neutrophil chemotaxis	0.033415	4
GO:0046427	Positive regulation of JAK-STAT cascade	0.033415	4
GO:0071345	Cellular response to cytokine stimulus	3.34E-02	4
GO:0030199	Collagen fibril organization	3.73E-02	5
GO:0060021	Palate development	3.73E-02	7
GO:0032733	Positive regulation of interleukin-10 production	3.75E-02	4
GO:0009617	Response to bacterium	3.75E-02	4
GO:0007267	Cell-cell signaling	3.85E-02	15
GO:0033209	Tumor necrosis factor-mediated signaling pathway	0.039078	9
GO:0002377	Immunoglobulin production	0.040081	3
GO:0042127	Regulation of cell proliferation	0.040087	12
GO:0001895	Retina homeostasis	0.040381	5
GO:0034220	Ion transmembrane transport	0.042804	13
GO:0051209	Release of sequestered calcium ion into cytosol	0.043637	5
GO:0050900	Leukocyte migration	0.046074	9
GO:0046718	Viral entry into host cells	0.046145	7
GO:0007159	Leukocyte cell-cell adhesion	0.046473	4
GO:0001816	Cytokine production	0.046473	4
GO:0042511	Positive regulation of tyrosine phosphorylation of Stat1 protein	0.047952	3
GO:0050868	Negative regulation of T cell activation	0.047952	3

Supplementary Table S2. KEGG pathways enriched among DEGs in the Zurich cohort.

**KEGG ID**	**Pathway**	***P*-Value**	**No. of Genes**
hsa05340	Primary immunodeficiency	1.59E-13	16
hsa04640	Hematopoietic cell lineage	1.27E-09	19
hsa04060	Cytokine-cytokine receptor interaction	1.54E-07	28
hsa04660	T cell receptor signaling pathway	2.54E-06	16
hsa04514	Cell adhesion molecules (CAMs)	1.27E-05	18
hsa05146	Amoebiasis	2.48E-05	15
hsa04512	ECM-receptor interaction	6.15E-05	13
hsa04064	NF-kappa B signaling pathway	6.15E-05	13
hsa04670	Leukocyte transendothelial migration	6.24E-05	15
hsa04650	Natural killer cell-mediated cytotoxicity	0.00012	15
hsa04630	Jak-STAT signaling pathway	0.000222	16
hsa04672	Intestinal immune network for IgA production	0.000243	9
hsa04510	Focal adhesion	0.000449	19
hsa04662	B cell receptor signaling pathway	0.000783	10
hsa05162	Measles	0.003052	13
hsa04610	Complement and coagulation cascades	0.00326	9
hsa04020	Calcium signaling pathway	0.005272	15
hsa04080	Neuroactive ligand-receptor interaction	0.005342	20
hsa05032	Morphine addiction	0.005433	10
hsa04727	GABAergic synapse	0.011449	9
hsa04530	Tight junction	0.013072	9
hsa04972	Pancreatic secretion	0.018953	9
hsa04611	Platelet activation	0.019112	11
hsa04713	Circadian entrainment	0.021278	9
hsa05322	Systemic lupus erythematosus	0.023094	11
hsa04151	PI3K-Akt signaling pathway	0.024577	21
hsa04723	Retrograde endocannabinoid signaling	0.029455	9
hsa05144	Malaria	0.031148	6

Supplementary Table S3. GO biological processes enriched among DEGs in Stavanger cohort.

GO ID	Biological Processes	*P*-Value	No of Genes
GO:0006936	Muscle contraction	1.74E-06	14
GO:0006508	Proteolysis	1.23E-05	30
GO:0006805	Xenobiotic metabolic process	1.04E-04	10
GO:0030049	Muscle filament sliding	2.69E-04	7
GO:0007596	Blood coagulation	1.70E-03	13
GO:0042742	Defense response to bacterium	2.69E-03	11
GO:0048246	Macrophage chemotaxis	3.30E-03	4
GO:0014059	Regulation of dopamine secretion	3.30E-03	4
GO:0008202	Steroid metabolic process	3.57E-03	6
GO:0006937	Regulation of muscle contraction	4.13E-03	4
GO:0030154	Cell differentiation	4.19E-03	22
GO:0030574	Collagen catabolic process	4.32E-03	7
GO:0030855	Epithelial cell differentiation	6.71E-03	7
GO:0044267	Cellular protein metabolic process	7.70E-03	9
GO:0009404	Toxin metabolic process	8.12E-03	3
GO:0007409	Axonogenesis	9.41E-03	8
GO:0022617	Extracellular matrix disassembly	9.92E-03	7
GO:0071880	Adenylate cyclase-activating adrenergic receptor signaling pathway	1.01E-02	4
GO:0042475	Odontogenesis of dentin-containing tooth	1.02E-02	6
GO:0001867	Complement activation, lectin pathway	1.12E-02	3
GO:0019229	Regulation of vasoconstriction	1.16E-02	4
GO:0045987	Positive regulation of smooth muscle contraction	0.013325371	4
GO:0007268	Chemical synaptic transmission	0.013666242	13
GO:0060054	Positive regulation of epithelial cell proliferation involved in wound healing	0.014687488	3
GO:0030326	Embryonic limb morphogenesis	0.015242947	5
GO:0071377	Cellular response to glucagon stimulus	0.015242947	5
GO:0055085	Transmembrane transport	0.015308417	13
GO:0042493	Response to drug	0.015768856	15
GO:0007204	Positive regulation of cytosolic calcium ion concentration	0.015802623	9
GO:0007586	Digestion	0.01766679	6
GO:0052697	Xenobiotic glucuronidation	0.018586107	3
GO:0030299	Intestinal cholesterol absorption	0.018586107	3
GO:0051967	Negative regulation of synaptic transmission, glutamatergic	0.018586107	3
GO:0030511	Positive regulation of transforming growth factor-beta receptor signaling pathway	0.019232513	4
GO:0003009	Skeletal muscle contraction	0.019232513	4
GO:0031069	Hair follicle morphogenesis	0.026365735	4

GO:0003341	Cilium movement	0.026365735	4
GO:0015721	Bile acid and bile salt transport	0.026365735	4
GO:0006081	Cellular aldehyde metabolic process	0.027509606	3
GO:0007275	Multicellular organism development	0.028275062	21
GO:0019228	Neuronal action potential	0.029016236	4
GO:0007616	Long-term memory	0.029016236	4
GO:0006958	Complement activation, classical pathway	0.032277394	7
GO:0042572	Retinol metabolic process	0.034723245	4
GO:0030449	Regulation of complement activation	0.034723245	4
GO:0030324	Lung development	0.036170886	6
GO:0051926	Negative regulation of calcium ion transport	0.037801532	3
GO:0030818	Negative regulation of cAMP biosynthetic process	0.037801532	3
GO:0030239	Myofibril assembly	0.037801532	3
GO:0006809	Nitric oxide biosynthetic process	0.037801532	3
GO:0001516	Prostaglandin biosynthetic process	0.043413459	3
GO:0090090	Negative regulation of canonical Wnt signaling pathway	0.043689104	9
GO:0007186	G-protein coupled receptor signaling pathway	0.044036265	31
GO:0002088	Lens development in camera-type eye	0.044283479	4
GO:0002930	Trabecular meshwork development	0.047540711	2
GO:1903027	Regulation of opsonization	0.047540711	2
GO:0030198	Extracellular matrix organization	0.04793664	10
GO:0050905	Neuromuscular process	0.049312268	3

Supplementary Table S4. KEGG pathways enriched among DEGs in the Stavanger cohort.

**KEGG ID**	**Pathway**	***P*-Value**	**No of Genes**
hsa00830	Retinol metabolism	3.44E-04	16
hsa04610	Complement and coagulation cascades	5.78E-04	19
hsa05204	Chemical carcinogenesis	1.55E-03	28
hsa00982	Drug metabolism - cytochrome P450	2.56E-03	16
hsa04976	Bile secretion	2.79E-03	18
hsa00980	Metabolism of xenobiotics by cytochrome P450	0.004141708	15
hsa05414	Dilated cardiomyopathy	0.008279868	13
hsa00983	Drug metabolism - other enzymes	0.008346719	13
hsa04080	Neuroactive ligand-receptor interaction	0.009468349	15
hsa04972	Pancreatic secretion	0.014087876	15
hsa00350	Tyrosine metabolism	0.01512502	16
hsa04916	Melanogenesis	0.020284892	9
hsa00140	Steroid hormone biosynthesis	0.021420157	19
hsa00590	Arachidonic acid metabolism	0.026041828	10
hsa04974	Protein digestion and absorption	0.034324215	13
hsa04540	Gap junction	0.034324215	9
hsa04024	cAMP signaling pathway	0.047122625	15

Supplementary Table S5. Enriched fractions of the 64 cell types in primary and matched TNBC tumors in the Zurich cohort.

Cell Type	Group	xCell Enrichment Scores for 64- Cell Types (mean ±SD)-Zurich Cohort		
		Primary Tumors (n=11)	Matched Metastatic Tumors (n=11)	*P*-Value
Adipocytes	Stromal	0.006 ± 0.004	0.013 ± 0.007	0.349
Astrocytes	Others	0.0 ± 0.0	0.008 ± 0.0045	0.079
B cells	Immune	0.008 ± 0.004	0.200 ± 0.081	<0.05
Basophils	Immune	0.018 ± 0.007	0.011 ± 0.006	0.481
CD4 T cells	Immune	0.002 ± 0.001	0.041 ± 0.017	<0.05
Central Memory CD4+ T cell	Immune	0.0 ± 0.0	0.031 ± 0.012	<0.05
Effector Memory CD4+ T cell	Immune	0.0 ± 0.0	0.036 ± 0.014	<0.05
CD4 memory T cells	Immune	0.0057 ± 0.0026	0.032 ± 0.015	0.107
CD4 naïve T cells	Immune	0.00087 ± 0.0005	0.027 ± 0.015	0.082
CD8 T cells	Immune	0.00036 ± 0.0003	0.026 ± 0.011	<0.05
Central Memory CD8+ T cell	Immune	0.0036 ± 0.001	0.028 ± 0.012	0.051
Effector Memory CD8+T cell	Immune	0.0032 ± 0.002	0.0032 ± 0.002	0.153
CD8 naïve T cells	Immune	0.00095 ± 0.0006	0.004 ± 0.0027	0.308
Common Lymphoid Progenitor	Stem	0.014 ± 0.005218	0.013 ± 0.0048	0.944
Common Myeloid Progenitor	Stem	0.00013 ± 8.954e-00	0.0005 ± 0.0003	0.287
Chondrocytes	Stromal	0.0031± 0.0023	0.01325 ± 0.005	0.119
Class-switched memory B cells	Immune	0.019 ± 0.008	0.062 ± 0.018	<0.05
Dendritic Cells	Immune	0.00016 ± 0.0001	0.003 ± 0.002	0.157
Endothelial cells	Stromal	0.012 ± 0.004	0.023 ± 0.009	0.271
Eosinophils	Immune	8.182e-005 ± 8.182e-005	8.182e-005 ± 8.182e-005	0.329
Epithelial cells	Others	0.014 ± 0.0036	0.071 ± 0.018	<0.05
Erythrocytes	Stem	1.818e-005 ± 1.818e-005	0.0 ± 0.0	0.329
Fibroblasts	Stromal	0.046 ± 0.01716	0.021± 0.009	0.221
Granulocyte Macrophage Progenitor	Stem	0.001 ± 0.001041	0.0025± 0.0025	0.633
HSC	Stem	0.168 ± 0.043	0.195 ± 0.046	0.683
Hepatocytes	Others	0.00054± 0.0003	0.00013±0.00013	0.238
Keratinocytes	Others	0.003 ± 0.0019	0.017 ± 0.009	0.167
Megakaryocyte Erythroid Progenitor	Stem	0.016 ± 0.005	0.008 ± 0.003	0.117

Multipotent Progenitor	Stem	0.0 ± 0.0	0.0 ± 0.0	
Mesenchymal Stem Cells	Stem	0.074 ± 0.024	0.063 ± 0.018	0.718
Macrophages	Immune	0.005 ± 0.002	0.001 ± 0.0005	0.070
Macrophage M1	Immune	0.002± 0.0015	0.004 ± 0.0014	0.282
Macrophage M2	Immune	0.005 ± 0.003	0.003 ± 0.0015	0.580
Memory B cells	Immune	0.0004 ±0.0004	0.068 ± 0.028	<0.05
Mesangial cells	Others	0.0057 ± 0.0016	0.008 ± 0.0027	0.379
Monocytes	Immune	0.0 ± 0.0	0.00089 ± 0.0005	0.138
Myocytes	Stromal	0.0107 ± 0.007629	0.0007545 ± 0.0007545	0.209
NK cells	Immune	0	0	
Natural killer T cells	Immune	0.0099 ± 0.0035	0.0167 ± 0.006	0.366
Neutrophils	Immune	0	0	
Neurons	Others	0.0003 ± 0.00016	0.0002 ± 0.000107	0.459
Osteoblasts	Stromal	0.0005 ± 0.0003	0.0 ± 0.0	0.152
Pericytes	Stromal	0.034 ± 0.011	0.0341 ± 0.009	0.987
Plasma cells	Immune	0.0005 ± 0.0004	0.01 ± 0.006	0.157
Platelets	Stem	0.0 ± 0.0	0.0019 ± 0.0012	0.149
Preadipocytes	Stromal	0.023 ± 0.007	0.02011 ± 0.009	0.755
Sebocytes	Others	8.182e-005 ± 5.012e-005	0.0051 ± 0.003	0.169
Skeletal muscle	Stromal	0.006 ± 0.006	0.001 ± 0.0004	0.397
Smooth muscle	Stromal	0.062 ± 0.019	0.098 ± 0.022	0.235
Gamma delta T cells	Immune	0.005 ± 0.0025	0.004 ± 0.002	0.688
Th1 cells	Immune	0.004 ± 0.003	0.002 ± 0.0015	0.549
Th2 cells	Immune	0.031 ± 0.012	0.014 ± 0.009	0.325
Regulatory T cells	Immune	0.0019 ± 0.001002	0.008 ± 0.003	0.078
Activated dendritic cells	Immune	0.0 ± 0.0	0.014 ± 0.007	0.063
Conventional dendritic cells	Immune	0.022 ± 0.007	0.046 ± 0.022	0.330
Immature dendritic cells	Immune	0.052 ± 0.011	0.081 ± 0.029	0.363
Lymphatic endothelial cells	Stromal	0.0016 ± 0.00083	0.004 ± 0.003	0.394
Microvascular endothelial cells	Stromal	0.009 ± 0.003	0.022 ± 0.008	0.167
Naïve B cells	Immune	0.002 ± 0.0014	0.040 ± 0.016	<0.05
Plasmacytoid cells	Immune	0.0 ± 0.0	0.009 ± 0.005	0.099
Pro-B cells	Immune	0.0011 ± 0.0011	0.012 ± 0.005	0.076
Mast cells	Immune	0.0 ± 0.0	0.0016 ± 0.0009	0.085
Megakaryocytes	Stem	0.0005 ± 0.0002	0.0009 ± 0.0006	0.566
Melanocytes	Others	0.0005 ± 0.0001	0.0002 ± 0.0001	0.048
**Immune score**		0.011 ± 0.0031	0.183 ± 0.0720	<0.05
**Stromal score**		0.032 ± 0.012	0.029 ± 0.012	0.870

Supplementary Table S6. Enriched fractions of the 64 cell types in metastatic and non-metastatic primary TNBC tumors in the Zurich cohort.

Cell Type	Group	xCell Enrichment Scores for 64- Cell Types (mean ±SD)-Zurich Cohort		
		Metastatic Primary Tumors (n=13)	Non-Metastatic Primary Tumors (n=18)	*P*-Value
Adipocytes	Stromal	0.00652 ± 0.004	0.0217 ± 0.009	0.215
Astrocytes	Others	0.0 ± 0.0	0.0 ± 0.0	
B cells	Immune	0.008 ± 0.0038	0.038 ± 0.0146	0.100
Basophils	Immune	0.0152 ± 0.024	0.031 ± 0.007	0.135
CD4 T cells	Immune	0.0018 ± 0.0008	0.0035 ± 0.0018	0.449
Central Memory CD4+T cell	Immune	0.0027 ± 0.0028	0.0018 ± 0.0013	0.737
Effector Memory CD4+T cell	Immune	0.0 ± 0.0	0.022 ± 0.008	<0.05
CD4 memory T cells	Immune	0.0048 ± 0.0023	0.0127 ± 0.005	0.203
CD4 naïve T cells	Immune	0.0009 ± 0.0005	0.0007 ± 0.0006	0.833
CD8 T cells	Immune	0.0015 ± 0.001	0.011 ± 0.005	0.098
Central Memory CD8+ T cell	Immune	0.003031± 0.001597	0.021 ± 0.006	<0.05
Effector Memory CD8+T cell	Immune	0.0 ± 0.0	0.002 ± 0.0012	0.160
CD8 naïve T cells	Immune	0.0008 ± 0.0005	0.006 ± 0.0018	<0.05
Common Lymphoid Progenitor	Stem	0.012 ± 0.005	0.018 ± 0.0047	0.429
Common Myeloid Progenitor	Stem	0.00012 ± 0.00007	0.00012 ± 0.00009	0.924
Chondrocytes	Stromal	0.003 ± 0.002	0.0079 ± 0.004	0.299
Class-switched memory B cells	Immune	0.0212 ± 0.007	0.033 ± 0.009	0.392
Dendritic Cells	Immune	0.00015 ± 0.00012	0.0045 ± 0.0012	<0.05
Endothelial cells	Stromal	0.0103 ± 0.0036	0.0309 ± 0.0092	0.077
Eosinophils	Immune	0.0 ± 0.0	0.0 ± 0.0	
Epithelial cells	Others	0.015 ± 0.003	0.021± 0.004	0.222
Erythrocytes	Stem	0.0 ± 0.0	0.0 ± 0.0	
Fibroblasts	Stromal	0.040 ± 0.015	0.088 ± 0.023	0.112
Granulocyte Macrophage Progenitor	Stem	0.0034 ± 0.0014	0.003 ± 0.0008	0.197
HSC	Stem	0.161 ± 0.037	0.192 ± 0.037	0.560
Hepatocytes	Others	0.0004 ± 0.0003	0.001 ± 0.0005	0.110
Keratinocytes	Others	0.0048 ± 0.0019	0.0018 ± 0.0006	0.105

Megakaryocyte Erythroid Progenitor	Stem	0.016 ± 0.004	0.012 ± 0.003	0.424
Multipotent Progenitor	Stem	0.0 ± 0.0	0.0 ± 0.0	
Mesenchymal Stem Cells	Stem	0.084 ± 0.022	0.053 ± 0.013	0.217
Macrophages	Immune	0.005 ± 0.002	0.013 ± 0.004	0.145
Macrophage M1	Immune	0.003 ± 0.0018	0.007 ± 0.003	0.278
Macrophage M2	Immune	0.0038 ± 0.0024	0.007 ± 0.002	0.274
Mast cells	Immune	0.00004 ± 0.00005	0.002 ± 0.0008	<0.05
Memory B cells	Immune	0.00036 ± 0.0003	0.006 ± 0.003	0.083
Mesangial cells	Others	0.0047 ± 0.014	0.0054 ± 0.0015	0.768
Monocytes	Immune	0.0 ± 0.0	0.004 ± 0.002	0.05
Myocytes	Stromal	0.009 ± 0.006	0.001 ± 0.0004	0.134
NK cells	Immune	0.0 ± 0.0	0.0 ± 0.0	
Natural killer T cells	Immune	0.0124 ± 0.004	0.015 ± 0.004	0.672
Neutrophils	Immune	0.0 ± 0.0	0.0 ± 0.0	
Neurons	Others	0.0003 ± 0.0001	0.0015 ± 0.0003	<0.05
Osteoblasts	Stromal	0.002 ± 0.0009	0.014 ± 0.005	0.076
Pericytes	Stromal	0.039 ± 0.010	0.030794 ± 0.006	0.500
Plasma cells	Immune	0.0006 ± 0.0003	0.007 ± 0.003	0.055
Platelets	Stem	0.0 ± 0.0	0.0 ± 0.0	
Preadipocytes	Stromal	0.021 ± 0.006	0.025 ± 0.005	0.641
Sebocytes	Others	0.0002 ± 0.00001	0.00006 ± 0.00004	0.181
Skeletal muscle	Stromal	0.0 ± 0.0	0.0 ± 0.0	
Smooth muscle	Stromal	0.058 ± 0.017	0.026 ± 0.010	0.105
Gamma delta T cells	Immune	0.005 ± 0.002	0.027 ± 0.006	<0.05
Th1 cells	Immune	0.0 ± 0.0	0.0 ± 0.0	
Th2 cells	Immune	0.026 ± 0.011	0.080 ± 0.019	<0.05
Regulatory T cells	Immune	0.002 ± 0.00009	0.0004 ± 0.0004	0.138
Activated dendritic cells	Immune	0.002 ± 0.001	0.047 ± 0.017	<0.05
Conventional dendritic cells	Immune	0.019 ± 0.006	0.0679 ± 0.012	<0.05
Immature dendritic cells	Immune	0.052 ± 0.010	0.085 ± 0.016	0.14
Lymphatic endothelial cells	Stromal	0.0013 ± 0.0007	0.008 ± 0.0034	0.119
Microvascular endothelial cells	Stromal	0.009 ± 0.022	0.031 ± 0.004	0.472
Naïve B cells	Immune	0.002 ± 0.0011	0.002 ± 0.001	0.967
Plasmacytoid cells	Immune	0.00003 ± 0.00003	0.005 ± 0.002	0.062
Pro-B cells	Immune	0.0009 ± 0.0009	0.008 ± 0.0025	<0.05
Megakaryocytes	Stem	0.0006 ± 0.0002	0.004 ± 0.001	0.025
Melanocytes	Others	0.0005 ± 0.0001	0.001 ± 0.0003	0.094
**Immune score**		0.011 ± 0.002	0.051 ± 0.017	0.057
**Stromal score**		0.028 ± 0.010	0.070 ± 0.020	0.109

Supplementary Table S7. Enriched fractions of the 64 cell types in primary and matched TNBC tumors in the Stavanger cohort.

Cell Type	Group	xCell Enrichment Scores for 64- Cell Types (mean ± SD)-Stavanger Cohort		
		**Primary Tumors (n=5)**	**Matched Metastatic Tumors (n=5)**	***P*-Value**
Adipocytes	Stromal	0.016 ± 0.011	0.0008 ± 0.0005	0.198
Astrocytes	Others	0.0 ± 0.0	0.0068 ± 0.005	0.181
B cells	Immune	0.002 ± 0.001	0.011 ± 0.011	0.453
Basophils	Immune	0.024 ± 0.017	0.023 ± 0.013	0.995
CD4 T cells	Immune	0.0 ± 0.0	0.0 ± 0.0	
Central Memory CD4+T cell	Immune	0.0 ± 0.0	0.0 ± 0.0	
Effector Memory CD4+T cell	Immune	0.0003 ± 0.0003	0.005 ± 0.0034	0.236
CD4 memory T cells	Immune	0.0001 ± 0.0001	0.0 ± 0.0	0.347
CD4 naïve T cells	Immune	0.0 ± 0.0	0.0 ± 0.0	
CD8 T cells	Immune	0.0 ± 0.0	0.0 ± 0.0	
Central Memory CD8+ T cell	Immune	0.00034 ± 0.00034	4.000e-005 ± 4.000e-005	0.406
Effector Memory CD8+T cell	Immune	0.0 ± 0.0	0.0 ± 0.0	
CD8 naïve T cells	Immune	0.0 ± 0.0	0.0013 ± 0.001	0.275
Common Lymphoid Progenitor	Stem	0.004 ± 0.004	0.015± 0.0069	0.218
Common Myeloid Progenitor	Stem	0.0002 ± 0.0002	0.0 ± 0.0	0.347
Chondrocytes	Stromal	0.0035 ± 0.0026	0.00264 ± 0.00264	0.820
Class-switched memory B cells	Immune	0.006 ± 0.002	0.002± 0.002	0.336
Dendritic Cells	Immune	0.0 ± 0.0	0.0001 ± 0.0001	0.347
Endothelial cells	Stromal	0.006 ± 0.004	0.015 ± 0.009	0.347
Eosinophils	Immune	0.0003 ± 0.0003	0.00156 ± 0.00156	0.442
Epithelial cells	Others	0.004 ± 0.003	0.01248 ± 0.009654	0.446
Erythrocytes	Stem	0.0 ± 0.0	0.0 ± 0.0	
Fibroblasts	Stromal	0.003 ± 0.003	0.03746 ± 0.01902	0.115
Granulocyte Macrophage Progenitor	Stem	0.00014 ± 0.00014	0.0 ± 0.0	0.346
HSC	Stem	0.045 ± 0.013	0.049 ± 0.018	0.846
Hepatocytes	Others	0.0006 ± 0.0006	0.003± 0.0027	0.403
Keratinocytes	Others	0.0005 ± 0.0003	0.008 ± 0.006	0.255

Megakaryocyte Erythroid Progenitor	Stem	0.0097 ± 0.005	0.007 ± 0.003	0.736
Multipotent Progenitor	Stem	0.0 ± 0.0	0.0 ± 0.0	
Mesenchymal Stem Cells	Stem	0.053 ± 0.023	0.034 ± 0.015	0.513
Macrophages	Immune	0.002 ± 0.0015	0.0014 ± 0.0006	0.912
Macrophage M1	Immune	0.002 ± 0.0015	0.001 ± 0.0005	0.561
Macrophage M2	Immune	0.0018 ± 0.0009	0.00052 ± 0.0002	0.233
Mast cells	Immune	0.004 ± 0.0018	2.000e-005 ± 2.000e-005	0.044
Memory B cells	Immune	0.0 ± 0.0	0.001 ± 0.0013	0.322
Mesangial cells	Others	0.004 ± 0.0024	0.0005 ± 0.0005	0.234
Monocytes	Immune	0.0 ± 0.0	0.0 ± 0.0	
Myocytes	Stromal	0.001 ± 0.0007	0.0044 ± 0.0043	0.473
NK cells	Immune	0.0 ± 0.0	0.0 ± 0.0	
Natural killer T cells	Immune	0.0046 ± 0.003	0.007 ± 0.005	0.6702
Neutrophils	Immune	0.0 ± 0.0	0.0 ± 0.0	
Neurons	Others	0.00024 ± 0.00011	6.000e-005 ± 6.000e-005	0.195
Osteoblasts	Stromal	0.002 ± 0.0014	0.00868 ± 0.005	0.307
Pericytes	Stromal	0.0 ± 0.0	0.01572 ± 0.012	0.232
Plasma cells	Immune	0.00578 ± 0.003723	0.0040 ± 0.0012	0.662
Platelets	Stem	0.0008 ± 0.0008	0.0059 ± 0.0012	0.007
Preadipocytes	Stromal	0.0071 ± 0.003420	0.0198 ± 0.01213	0.343
Sebocytes	Others	0.0006 ± 0.0004	0.00306 ± 0.0026	0.380
Skeletal muscle	Stromal	0.0 ± 0.0	0.00144 ± 0.001	0.346
Smooth muscle	Stromal	0.01926 ± 0.01184	0.042 ± 0.017	0.303
Gamma delta T cells	Immune	2.000e-005 ± 2.000e-005	0.0 ± 0.0	0.347
Th1 cells	Immune	0.007 ± 0.005	0.004 ± 0.002	0.590
Th2 cells	Immune	0.033 ± 0.015	0.016 ± 0.011	0.392
Regulatory T cells	Immune	0.0 ± 0.0	0.00098 ± 0.0009	0.311
Activated dendritic cells	Immune	0.003 ± 0.002	0.0019 ± 0.0019	0.711
Conventional dendritic cells	Immune	0.0 ± 0.0	0.008 ± 0.003	0.0477
Immature dendritic cells	Immune	0.019 ± 0.007	0.052 ± 0.019	0.165
Lymphatic endothelial cells	Stromal	0.0004 ± 0.0003	0.0015 ± 0.002	0.503
Microvascular endothelial cells	Stromal	0.003 ± 0.002	0.008 ± 0.006	0.419
Naïve B cells	Immune	0.0008 ± 0.0006	0.002 ± 0.002	0.553
Plasmacytoid cells	Immune	0.0 ± 0.0	0.0 ± 0.0	

Pro-B cells	Immune	0.0 ± 0.0	0.0021 ± 0.0013	0.142
Megakaryocytes	Stem	0.002 ± 0.002	0.001 ± 0.0009	0.654
Melanocytes	Others	0.001 ± 0.0006	0.002 ± 0.001	0.755
**Immune score**		0.003 ± 0.0010	0.011 ± 0.007	0.308
**Stromal score**		0.013 ± 0.008	0.027 ± 0.013	0.384
